# Supplementary material for: Development of the Acoustic Comfort Assessment Scale (ACAS-12): Psychometric properties, validity evidence and back-translation between Spanish and English
Source: PLoS One. 2023 Feb 7;18(2):e0281534. doi: 10.1371/journal.pone.0281534 (PMC9904492; doi:10.1371/journal.pone.0281534)
Supplement: S4 File — (DOCX) [file pone.0281534.s004.docx]

# S2 File. ACAS-12 scale

**S2 File.** **Acoustic Comfort Assessment Scale (*ACAS-12*) in Spanish (native language) and English**

|  | | **English** | | | **Español** | | |
| --- | --- | --- | --- | --- | --- | --- | --- |
| **Question statement** | | Concentrate on what you are hearing now. Take a minute to perceive the environment and sound of this place.  Next, could you please describe the acoustic environment you are hearing in this place from your point of view? | | | Ahora concéntrese en lo que está usted escuchando. Tómese un minuto en percibir el ambiente y el sonido de este lugar.  A continuación ¿Nos podría describir por favor, el ambiente sonoro que usted está escuchando en este lugar desde su punto de vista? | | |
| **Help** | | Use the adjective pairs listed below for this. Each adjective pair refers to the same aspect; one is positive and the other is negative. You should choose a number from 1 to 5 to best reflect your opinion. So:   - 1 would indicate that the adjective on the left describes the acoustic environment you are hearing very well. - 2 would indicate that the adjective on the left describes it well, but not as well as 1. - 3 would indicate that you are not sure which of the two adjectives (the one on the right or left) would be more appropriate - 5 would indicate that the adjective on the right describes acoustic environment very well. - 4 would indicate that the adjective on the right describes it well, but not as well as 5. | | | Use para ello los pares de adjetivos que aparecen más abajo. Cada par de adjetivos se refiere al mismo aspecto, uno de ellos en sentido positivo y otro en negativo. Usted debe elegir un número del 1 al 5 que mejor refleje su opinión. De este modo:   - 1 indicaría que el adjetivo de la izquierda describe muy bien el ambiente sonoro que está escuchando. - 2 indicaría que lo describe bien, pero no tan bien como el 1. - 3 indicaría que no está seguro de cual de los dos acjetivos (el de la derecha o izquierda) sería el más apropiado. - 5 indicaría que el adjetivo de la derecha describe muy bien el ambiente sonoro que está escuchando. - 4 indicaría que el adjetivo de la derecha describe bien el ambiente sonoro, pero no tan bien como el 5. | | |
| **Bipolar Adjectives** | **1** | unpleasant | 1 2 3 4 5 | pleasant | desagradable | 1 2 3 4 5 | agradable |
|  | **2** | stressful | 1 2 3 4 5 | relaxing | estresante | 1 2 3 4 5 | relajante |
|  | **3** | noisy | 1 2 3 4 5 | peaceful, calm | ruidoso | 1 2 3 4 5 | tranquilo |
|  | **4** | confusing, chaotic | 1 2 3 4 5 | clear, accurate | caótico, confuso | 1 2 3 4 5 | claro, nítido |
|  | **5** | boring | 1 2 3 4 5 | fun | aburrido | 1 2 3 4 5 | divertido |
|  | **6** | artificial | 1 2 3 4 5 | natural | artificial | 1 2 3 4 5 | natural |
|  | **7** | monotonous | 1 2 3 4 5 | lively | monótono | 1 2 3 4 5 | vibrante, animado |
|  | **8** | hinders conversation | 1 2 3 4 5 | facilitates conversation | dificulta conversación | 1 2 3 4 5 | facilita conversación |
|  | **9** | no informative | 1 2 3 4 5 | informative | no informativo | 1 2 3 4 5 | informativo |
|  | **10** | intermittent | 1 2 3 4 5 | uninterrupted | discontinuo | 1 2 3 4 5 | continuo |
|  | **11** | unknown | 1 2 3 4 5 | familiar | desconocido | 1 2 3 4 5 | familiar |
|  | **12** | inappropriate for the surroundings | 1 2 3 4 5 | appropriate for the surroundings | inapropiado con su entorno | 1 2 3 4 5 | apropiado con su entorno |
