# Supplementary material for: Development of the Acoustic Comfort Assessment Scale (ACAS-12): Psychometric properties, validity evidence and back-translation between Spanish and English
Source: PLoS One. 2023 Feb 7;18(2):e0281534. doi: 10.1371/journal.pone.0281534 (PMC9904492; doi:10.1371/journal.pone.0281534)
Supplement: Attachment — Submitted filename: Response to Reviewers.docx [file pone.0281534.s005.docx]

**Rebuttal letter**

We sincerely appreciate the reviewer's positive comments that contribute to the improvement of our manuscript. In the overall, we have taken into account all his/her suggestions. Below we briefly comment on the changes we have made following the suggestions we have considered more relevant and justify those we have not integrated into the manuscript.

In the first suggestion of the reviewer, he/she proposes referencing the indices mentioned in the methods section. The specific references have been included:

- new reference **[38]**: **Costelo & Osborne (2005**) in lines 302 and 306; and
- new reference **[43]**: **Osborne (2014)** in lines 310, 314, 316 and 318.

Regarding the second suggestion, we believe that the results section describes and interprets the values of the indexes whose “cut off” points have already been explained or indicated in the methodology section. We have not found any mention of re-specification of the “cut offs”.

On the other hand, we have considered the reviewer's third suggestion. On that sense the results concerning reliability and internal consistence (lines 418 to 424) have been moved to the end (lines 482-488), once the unidimensionality of the scale has been addressed.

Finally, the reviewer suggests the possibility of testing the invariance of the results according to variables of interest. Among the variables collected in our study, we consider that there are no variables that allow this type of analysis to be carried out. One possibility would be to analyzing invariance according to the sex of the participants. But there is no scientific evidence that would allow us to assess a differential behavior between men and women in the soundscape. We believe that it would be more relevant to test the scale invariance when it is applied for soundscapes with different characteristics. For example, to compare urban and natural soundscapes. Based on the reviewer's suggestion we will consider this option in our future evaluations in which we will consider other soundscape contexts, urban versus rural, technological versus natural, etc.

We reiterate our thanks to the reviewer for his/her comments and suggestions, and hope that we have managed to respond to his/her considerations.
