# Supplementary material for: Development of the Acoustic Comfort Assessment Scale (ACAS-12): Psychometric properties, validity evidence and back-translation between Spanish and English
Source: PLoS One. 2023 Feb 7;18(2):e0281534. doi: 10.1371/journal.pone.0281534 (PMC9904492; doi:10.1371/journal.pone.0281534)
Supplement: Attachment — Submitted filename: Response to Reviewers.docx [file pone.0281534.s007.docx]

**Rebuttal letter 2**

We sincerely appreciate the reviewer's positive comments that contribute to the improvement of our manuscript.

The comments and suggestions of reviewer #2 have been followed by correcting and clarifying the text of the manuscript. Some additional clarifications are:

A. At the request of reviewer #2, some **references** have been added in the text to support the assessments of the results (lines 346, 374, 395, 411). They had not been added as previous version of manuscript because they correspond to conventional values that either appeared in the references cited methodological description or in classical manuals, such as the one added to the references [new 56].
B. In response to the comment about **Kappa index valuation**: The authors know that the magnitude of the kappa values can be influenced by two conditions: 1) the codes are equiprobable or their probabilities vary, and 2) whether the marginal probabilities for the two observers are similar or different. However, to take a point of reference, we have considered Cohen's standard criteria, which are reflected in many psychometrics manuals (e.g., Barbero-García, Vila-Abad, Holgado, 2015 [55]), introducing in the text of the article a nuance referring to the value 0.692 by considering it as “**substantial”**: "The level of agreement obtained in both stages of translation was substantial (kappa = 0.692 in stage 1 {ES>EN}) and high (0.846 in stage 2 {EN>ES}) [53-55]".
C. In response to the comment about the **result of MAP and parallel test**: For the factor analyses, the FACTOR program [39-40] is used to analyse the correlation matrix of the ACAS-12 items. Based on the theory developed by Velicer [41], this program provides the result of the MAP and parallel tests. This is a proposal of the number of factors to be retained. In our case, as indicated in the text (line 420), the result is a single factor.
D. The reviewer asks us whether the **same group of participants** was used for the **exploratory and confirmatory factor analysis**, and the answer is yes. However, we would like to inform him of the reasons for this approach. Our a priori hypothesis is the existence of a single factor that is explained by the set of twelve facets of acoustic comfort as assessed by the 12 items of the ACAS, therefore, the main analysis is confirmatory factorisation, e.g., that the empirically observed data fit the proposed theoretical model. However, certain correlation matrix analysis procedures offered by the FACTOR program are used to provide further evidence of the timeliness of the factorisation (determinant close to zero, KMO > 0.80, and significant Bartlet's test of sphericity), of the assumption of one-dimensionality (MAP and Parallel tests, and criterion of the eigenvalue of the first factor greater than three times the eigenvalue of the second factor), and to obtain the communalities (h^2^), necessary for the calculation of the omega reliability index. Hence, the analyses of both factorisation processes come from the same group of participants.

We reiterate our thanks to the reviewer for his/her comments and suggestions, and hope that we have managed to respond to his/her considerations.
