# Supplementary material for: Development of the Acoustic Comfort Assessment Scale (ACAS-12): Psychometric properties, validity evidence and back-translation between Spanish and English
Source: PLoS One. 2023 Feb 7;18(2):e0281534. doi: 10.1371/journal.pone.0281534 (PMC9904492; doi:10.1371/journal.pone.0281534)
Supplement: Attachment — Submitted filename: Response to Reviewers.docx [file pone.0281534.s008.docx]

**Rebuttal letter 2-bis**

We sincerely appreciate the reviewer's positive comments that contribute to the improvement of our manuscript.

The comments and suggestions of reviewer #2 have been followed by correcting and clarifying the text of the manuscript:

| ***Comments and suggestions of reviewer #2*** | ***Authors' response*** |
| --- | --- |
| **Abstract** |  |
| - Line 23: please change “assessment of human perception” to “human perception assessment” | DONE |
| - Please be consistent: there are the terms five-point and 2-point and 3-point. Kindly use the full word or the number throughout. | all the manuscript done and revised |
| - Line 35: kindly change: “The back-translation technique has been used for writing the English version.” to “the back-translation technique has been applied to create the English version” | DONE |
| - L35-36: You mention that “The results highlight the good psychometric properties of the ACAS 12 scale” but no results have been presented in the abstract. I would suggest presenting some of your findings in the abstract to back-up this argument. | **Added**: *"…(Cronbach’s alpha 0.91 and composite reliability 0.90)”* |
| - Line 36: “factorial solution possible”; please delete the word possible from this sentence | DONE |
| - Line 37: please change the word “around” to “approximately” | DONE |
| - L36: Current factor analysis techniques are mostly based on Goodness of fit statistics (i.e., RMSEA, CFI, TLI, etc.). Kindly provide these in the abstract to reinforce your argument that a one-factor solution is the most appropriate one. | **Added**: "*The absolute and incremental fit indices were above the minimum reference value of 0.90 and the residual-based indices showed values close to suitability (SRMR=0.057; RMSEA=0.042This)*.” |
| **Text** |  |
| - Line 43: “This is why” please change to “this the reason for which” | DONE |
| - Line 43: “have been mainly focused” please delete the word “been” | DONE |
| - Lines 49-50: “The enhancement of comfort, in general, and acoustic comfort of urban spaces, in particular, entails considering them places for the enjoyment and relaxation of the people who use them” please rephrase, meaning not clear. | Clarified: the sentence has been deleted |
| - Line 54: please change the word “proposed” to “suggested” | DONE |
| - Line 67-68: “5) evaluation based on triangulation applying different methods” please rephrase meaning not clear. | **Clarified**: “*5) evaluation based on different methods of assessment (triangulation)”.* |
| - Line 88: “Its applies” please correct | DONE |
| - Line 95: “ACAS-12 is focus” please correct | DONE |
| - Line 98: please change word “where” to “in which” | DONE |
| - Line 128: please change to “obtained” | DONE |
| - Line 134: sentence refers to past or in general? If in past, then kindly use past tense. | DONE |
| - Line 152: please change the word “would” to “could” | DONE |
| - Line 157: kindly rephrase; it is very difficult to understand the meaning of this sentence. | Rewritten the first part of the first paragraph of methods section |
| - Line 346: please provide a reference about the kappa coefficient (why kappa=0.692 is characterised as high?) | See **RESPONSE A** (NEW references [53, 54,55]) and **RESPONSE B**. |
| - Line 373: “casts doubt” kindly rephrase | Rewritten |
| - Line 374: please provide relevant reference | See **RESPONSE A**  NEW reference [56]. |
| - Table 3: please indicate what 1, 2, and 3 stand for | DONE in the footer |
| - Line 395: please provide reference | See **RESPONSE A** NEW reference [56]. |
| - Table 4: kindly provide in the footnotes what numbers 1-5 mean | DONE in the footer |
| - Line 411: kindly provide a reference why percentages >15% constitute floor effect | See **RESPONSE A** NEW reference [57]. |
| **Major** |  |
| - Line 430: where can we find the results of the MAP and the parallel test? | See **RESPONSE C**  NEW references [58,59]. |
| - Line 436: did you use the same dataset to perform the CFA? | See **RESPONSE D** |

Some additional clarifications (RESPONSES) are:

- **RESPONSE A**: At the request of reviewer #2, some **references** have been added in the text to support the assessments of the results (lines 346, 374, 395, 411). They had not been added as previous version of manuscript because they correspond to conventional values that either appeared in the references cited methodological description or in classical manuals, such as the one added to the references [new 56].
- **RESPONSE B:** In response to the comment about **Kappa index valuation**: The authors know that the magnitude of the kappa values can be influenced by two conditions: 1) the codes are equiprobable or their probabilities vary, and 2) whether the marginal probabilities for the two observers are similar or different. However, to take a point of reference, we have considered Cohen's standard criteria, which are reflected in many psychometrics manuals (e.g., Barbero-García, Vila-Abad, Holgado, 2015 [55]), introducing in the text of the article a nuance referring to the value 0.692 by considering it as “**substantial”**: "The level of agreement obtained in both stages of translation was substantial (kappa = 0.692 in stage 1 {ES>EN}) and high (0.846 in stage 2 {EN>ES}) [53-55]".
- **RESPONSE C:** In response to the comment about the **result of MAP and parallel test**: For the factor analyses, the FACTOR program [39-40] is used to analyse the correlation matrix of the ACAS-12 items. Based on the theory developed by Velicer [41], this program provides the result of the MAP and parallel tests. This is a proposal of the number of factors to be retained. In our case, as indicated in the text (line 420), the result is a single factor.
- **RESPONSE D:** The reviewer asks us whether the **same group of participants** was used for the **exploratory and confirmatory factor analysis**, and the answer is yes. However, we would like to inform him of the reasons for this approach. Our a priori hypothesis is the existence of a single factor that is explained by the set of twelve facets of acoustic comfort as assessed by the 12 items of the ACAS, therefore, the main analysis is confirmatory factorisation, e.g., that the empirically observed data fit the proposed theoretical model. However, certain correlation matrix analysis procedures offered by the FACTOR program are used to provide further evidence of the timeliness of the factorisation (determinant close to zero, KMO > 0.80, and significant Bartlet's test of sphericity), of the assumption of one-dimensionality (MAP and Parallel tests, and criterion of the eigenvalue of the first factor greater than three times the eigenvalue of the second factor), and to obtain the communalities (h^2^), necessary for the calculation of the omega reliability index. Hence, the analyses of both factorisation processes come from the same group of participants.

In addition, a revision of the English language has been carried out.

We reiterate our thanks to the reviewer for his/her comments and suggestions, and hope that we have managed to respond to his/her considerations.
