# Supplementary material for: Development of the Acoustic Comfort Assessment Scale (ACAS-12): Psychometric properties, validity evidence and back-translation between Spanish and English
Source: PLoS One. 2023 Feb 7;18(2):e0281534. doi: 10.1371/journal.pone.0281534 (PMC9904492; doi:10.1371/journal.pone.0281534)
Supplement: Attachment — Submitted filename: Reviewer Comments.docx [file pone.0281534.s006.docx]

Thank you for the opportunity to review the work entitled "Development of the Acoustic Comfort Assessment Scale (ACAS-12): Psychometric properties, validity evidence and back-translation between Spanish and English".

You have done a really good job but there are a couple of things that would be great to address.

**Abstract**

•       Line 23: please change “assessment of human perception” to “human perception assessment”

•       Please be consistent: there are the terms five-point and 2-point and 3-point. Kindly use the full word or the number throughout.

•       Line 35: kindly change: “The back-translation technique has been used for writing the English version.” to “the back-translation technique has been applied to create the English version”

•       You mention that “The results highlight the good psychometric 36 properties of the ACAS 12 scale” but no results have been presented in the abstract. I would suggest presenting some of your findings in the abstract to back-up this argument.

•        Line 36: “factorial solution possible”; please delete the word possible from this sentence

•       Line 37: please change the word “around” to “approximately”

•       Current factor analysis techniques are mostly based on Goodness of fit statistics (i.e., RMSEA, CFI, TLI, etc.). Kindly provide these in the abstract to reinforce your argument that a one-factor solution is the most appropriate one.

**Text**

•       Line 43: “This is why” please change to “this the reason for which”

•       Line 43: “have been mainly focused” please delete the word “been”

•       Lines 49-50: “The enhancement of comfort, in general, and acoustic comfort of urban spaces, in particular, entails considering them places for the enjoyment and relaxation of the people who use them” please rephrase, meaning not clear.

•       Line 54: please change the word “proposed” to “suggested”

•       Line 67-68: “5) evaluation based on triangulation applying different methods” please rephrase meaning not clear.

•       Line 88: “Its applies” please correct

•       Line 95: “ACAS-12 is focus” please correct

•       Line 98: please change word “where” to “in which”

•       Line 128: please change to “obtained”

•       Line 134: sentence refers to past or in general? If in past, then kindly use past tense.

•       Line 152: please change the word “would” to “could”

•       Line 157: kindly rephrase; it is very difficult to understand the meaning of this sentence.

•       Line 346: please provide a reference about the kappa coefficient (why kappa=0.692 is characterised as high?)

•       Line 373: “casts doubt” kindly rephrase

•       Line 374: please provide relevant reference

•       Table 3: please indicate what 1, 2, and 3 stand for

•       Line 395: please provide reference

•       Table 4: kindly provide in the footnotes what numbers 1-5 mean

•       Line 411: kindly provide a reference why percentages >15% constitute floor effect

**Major**

- Line 430: where can we find the results of the MAP and the parallel test?

•       Line 436: did you use the same dataset to perform the CFA?
